# Assessing the Association of Pain Intensity Scales on Quality of Life in Elderly Patients with Chronic Pain: A Nursing Approach

**DOI:** 10.3390/healthcare12202078

**Published:** 2024-10-18

**Authors:** Abdulaziz M. Alodhialah, Ashwaq A. Almutairi, Mohammed Almutairi

**Affiliations:** 1 Department of Medical Surgical Nursing, College of Nursing, King Saud University, Riyadh 11421, Saudi Arabia; mohalmutairi@ksu.edu.sa; 2 School of Nursing & Midwifery, Monash University, Clayton, VIC 3168, Australia; ashwaq.almutairi@monash.edu

**Keywords:** chronic pain, elderly, quality of life, pain assessment, nursing care

## Abstract

Background: Chronic pain is prevalent among the elderly and significantly affects their quality of life (QoL). Pain intensity scales are crucial tools in evaluating the severity of pain and tailoring management strategies. This study investigates the relationship between various pain intensity scales and QoL among elderly patients with chronic pain, highlighting the implications for nursing practice. Methods: A cross-sectional study was conducted with 150 elderly patients (aged 65 and above) in Riyadh, Saudi Arabia. Participants were assessed using the Numeric Rating Scale (NRS), Visual Analog Scale (VAS), and McGill Pain Questionnaire (MPQ) alongside the 36-Item Short-Form Health Survey (SF-36) to evaluate QoL. Data analysis involved Pearson correlation and multiple regression to explore the association of pain intensity on QoL. Results: All pain scales showed significant negative correlations with QoL. The MPQ exhibited a significant association, suggesting its comprehensive nature captures the multidimensional association of pain more effectively. Regression analysis identified pain intensity, age, and duration of chronic pain as significant predictors of reduced QoL. Conclusions: The findings emphasize the importance of selecting appropriate pain assessment tools that reflect the complex nature of pain in elderly patients. Implementing comprehensive pain assessments like the MPQ can enhance individualized care strategies and potentially improve the QoL in this population. This study underscores the role of nurses in optimizing pain management approaches tailored to the elderly.

## 1. Introduction

Chronic pain is a pervasive public health challenge that disproportionately affects the elderly, significantly associating their physical capabilities, mental health, and overall quality of life [1]. This issue is of particular concern as more than half of the senior population globally suffers from chronic pain, which not only diminishes their daily functioning and independence but also escalates their reliance on healthcare services and caregivers [2]. The effective management and treatment of chronic pain in this demographic are crucial, not only for the direct benefit of the individuals afflicted but also for alleviating the broader economic pressures on healthcare systems and societal structures [3,4].

As the global demographic shifts toward an aging population, with projections indicating nearly 2 billion individuals aged 65 and over by 2050, the imperative to address age-associated conditions like chronic pain becomes increasingly urgent [5]. Chronic pain among the elderly is inherently complex, often exacerbated by the co-occurrence of other chronic conditions such as diabetes, arthritis, and cardiovascular diseases [6]. These comorbidities can obscure the clinical picture and complicate both the assessment and management of pain, rendering traditional pain management strategies less effective and often inappropriate [7].

Underreporting and undertreatment of pain in the elderly stem from various factors. Common misconceptions that pain is a natural part of aging, fears of invasive treatments, and the potential side effects of pain medication contribute to a hesitancy to seek or report pain [8]. Cognitive impairments, prevalent in this age group, further complicate communication about pain levels, making accurate assessment challenging. Additionally, elderly patients are particularly susceptible to adverse effects from pharmacological treatments, including increased risks of falls, cognitive dysfunction, and gastrointestinal disturbances [9]. These issues often lead to a preference for conservative management strategies that may not provide sufficient pain relief, thus perpetuating a cycle of suffering and functional decline [10].

Accurate assessment of pain intensity is critical for effective pain management. It not only informs the choice of treatment strategies but also significantly influences outcomes related to pain relief and functional improvement [11,12]. However, pain assessment in the elderly is hindered by factors such as sensory deficits, cognitive decline, and psychological conditions, all of which can alter pain perception and self-reporting [13]. Consequently, there exists a pronounced need for pain assessment tools that are both sensitive to and specifically designed for the complexities presented by the elderly population [14].

In clinical settings, pain scales are essential tools used to measure pain intensity. These scales enable patients to quantify and communicate their pain levels, which, in turn, guide clinical decision-making and treatment strategies [15]. However, the effectiveness of these tools varies considerably among older adults due to the diverse ways in which they experience and articulate pain [16]. The primary challenge for healthcare providers is to select and utilize pain assessment tools that can accurately capture the multidimensional nature of pain as experienced by elderly patients, ensuring that the data obtained accurately reflect their pain experience and effectively guide appropriate management strategies [17].

The economic burden of chronic pain is substantial, encompassing both direct costs, such as increased healthcare utilization, and indirect costs, including lost productivity and caregiver burden [18]. With the anticipated increase in the elderly population, these costs are expected to rise significantly, underscoring the urgency to develop effective pain management strategies that can mitigate the association of chronic pain in both individuals and the broader society [19]. The development and implementation of such strategies will not only improve the quality of life for the elderly but will also provide significant economic relief by reducing the demand for healthcare resources and supporting more sustainable healthcare practices [20].

### 1.1. Aim of the Study

The primary aim of this study is to evaluate the effectiveness of various pain assessment tools in reflecting the true extent of pain experienced by elderly patients and their association with quality of life. This study seeks to identify which pain assessment methodologies are most suitable for elderly patients, considering the unique challenges this population faces in accurately communicating and managing their pain. By determining the most effective tools for assessing pain in this demographic, the research aims to guide healthcare professionals in selecting appropriate strategies that enhance pain management and improve the quality of life for elderly individuals.

### 1.2. Research Questions

1. How do different pain assessment tools compare in their effectiveness in accurately measuring pain intensity and its association with the quality of life in elderly patients with chronic pain?
2. Which pain assessment methodologies are most correlated with improved pain management outcomes and enhanced quality of life among the elderly?

## 2. Materials and Methods

### 2.1. Study Design

This study employed a cross-sectional descriptive design to assess the association of pain intensity scales on the quality of life in elderly patients with chronic pain from a nursing perspective. The cross-sectional approach was chosen to capture data at a single point in time, allowing for the examination of the relationship between pain intensity, as measured by various scales, and quality of life among the elderly population. This design is particularly suitable for understanding patterns and associations in the specified patient population.

### 2.2. Setting

The study was conducted in various secondary healthcare settings across Riyadh, Saudi Arabia. These settings included outpatient clinics, geriatric care units, and pain management clinics in multiple hospitals within the region. The chosen settings represent a diverse range of healthcare facilities where elderly patients commonly receive care for chronic pain, thereby ensuring the generalizability of the study findings across different healthcare environments.

### 2.3. Sample and Sampling

The target population for this study comprised elderly patients aged 65 years and older who were diagnosed with chronic pain and were receiving treatment in various secondary healthcare settings across Riyadh, Saudi Arabia. Chronic pain was defined as pain persisting for three months or more, regardless of the underlying cause. The sample was intended to represent a diverse group of elderly patients, including those with varying degrees of pain severity, different chronic pain conditions, and diverse demographic backgrounds.

### 2.4. Inclusion and Exclusion Criteria

To ensure that the sample accurately reflected the population of interest, the following inclusion and exclusion criteria were applied:

- Inclusion Criteria:

1. Patients aged 65 years and older;
2. Patients diagnosed with chronic pain lasting three months or more;
3. Patients currently receiving treatment for chronic pain in one of the selected secondary healthcare settings;
4. Patients able to communicate effectively in either Arabic or English;
5. Patients capable of providing informed consent independently or through a legally authorized representative.

- Exclusion Criteria:

1. Patients with severe cognitive impairments that would prevent them from accurately reporting their pain levels or completing the questionnaire (e.g., advanced dementia);
2. Patients experiencing acute pain episodes or who were hospitalized for acute conditions at the time of the study;
3. Patients with terminal illnesses where end-of-life care was the primary focus rather than chronic pain management.

### 2.5. Sampling Method

A purposive sampling method was employed to select participants who met the inclusion criteria. Purposive sampling is a non-probability sampling technique often used in qualitative research to identify and select individuals or groups of individuals who are especially knowledgeable about or experienced with a phenomenon of interest. In this study, purposive sampling was chosen to ensure that the sample included elderly patients who were actively experiencing chronic pain and were engaged in ongoing pain management within the healthcare system.

The sampling process was facilitated by nursing staff in the participating healthcare settings. Nurses who were familiar with the patients’ medical histories and treatment plans identified eligible participants and introduced the study to them.

### 2.6. Sample Size Determination

The sample size was determined based on the study’s objectives, the expected variability in pain intensity and quality-of-life scores, and practical considerations such as time and resource constraints. Using the G*Power software (version 3.1.9.7) for sample size calculation [21], a target sample size of 150 participants was deemed sufficient to detect statistically significant relationships between pain intensity scores and quality-of-life outcomes.

The calculation was based on the following assumptions [22]:

- A medium effect size (Cohen’s d = 0.3) for the relationship between pain intensity and quality of life;
- A power level of 0.80, indicating an 80% chance of detecting a true effect if it exists;
- A significance level of 0.05 (alpha), which is the standard threshold for determining statistical significance.

Given the expected challenges in recruiting elderly patients, such as potential health issues or transportation difficulties, the sample size was adjusted slightly higher to account for possible dropouts or incomplete data.

### 2.7. Recruitment Process

Recruitment took place in various secondary healthcare settings, including outpatient clinics, geriatric care units, and pain management clinics within hospitals in Riyadh. Nurses working in these settings played a crucial role in identifying eligible patients and inviting them to participate in the study.

Once potential participants were identified, they were approached by the research team or the assisting nursing staff. Participants were provided with a detailed explanation of the study, including its purpose, procedures, potential risks, and benefits. Those who agreed to participate were asked to sign an informed consent form.

Special consideration was given to ensuring that participants fully understood the study and their role in it. For participants with mild cognitive impairments or those who required assistance with reading or comprehension, the research team offered additional support, such as reading the consent form aloud or providing simplified explanations.

### 2.8. Data Collection Tool

Data were collected using a structured questionnaire that comprised three main sections: demographic information, pain intensity scales, and quality-of-life measurement.

1. Demographic Information:

This study is designed to collect essential background data on participants, providing context for the analysis of pain intensity and quality of life. This section of the questionnaire includes questions about the participants’ age, sex, duration of chronic pain, comorbid conditions, and current pain management strategies. Age is recorded in years, while sex is captured as male or female. Participants are asked to specify how long they have been experiencing chronic pain, expressed in months or years, and to indicate any additional health issues, such as diabetes or cardiovascular disease, through a checklist of common comorbidities. Additionally, participants describe their current pain management approaches, including both pharmacological treatments (e.g., NSAIDs, opioids) and non-pharmacological methods (e.g., physical therapy, alternative therapies).

2. Numeric Rating Scale (NRS)

The Numeric Rating Scale (NRS) is designed to measure the intensity of a patient’s pain on a simple 0 to 10 scale, where 0 represents “no pain” and 10 signifies “the worst pain imaginable.” Developed in the 1970s as an easy-to-use tool, the NRS allows patients to quickly communicate their pain levels, making it particularly useful in clinical settings and for populations with limited literacy or cognitive ability. The score provided by the patient is directly used to assess pain severity, with higher scores indicating more intense pain. The NRS has demonstrated high reliability, with test–retest correlations often exceeding 0.85, making it a valid tool for chronic pain assessment, especially in elderly patients.

3. Visual Analog Scale (VAS)

The Visual Analog Scale (VAS), introduced by Aitken in 1969, is a widely used tool for measuring pain intensity in a more nuanced way. It consists of a 10 cm horizontal or vertical line anchored by “no pain” on the left and “worst imaginable pain” on the right. Patients mark a point on the line that best represents their pain, with the distance from the left end measured to determine their pain score, typically on a 0 to 100 scale. The VAS is appreciated for its sensitivity in detecting subtle changes in pain intensity, though it requires clear patient understanding for accurate use. It has proven reliable, with test–retest correlations often above 0.90, although it can be challenging for patients with cognitive impairments.

4. McGill Pain Questionnaire (MPQ)

The McGill Pain Questionnaire (MPQ), developed by Dr. Ronald Melzack and Dr. Patrick Wall in 1975 [23], is a comprehensive tool that assesses both the intensity and quality of pain across sensory, affective, and evaluative dimensions. The MPQ includes 78 descriptive terms categorized into groups alongside a Present Pain Intensity (PPI) scale ranging from 0 (no pain) to 5 (excruciating pain). This multidimensional assessment provides a detailed picture of the patient’s pain experience, offering scores that reflect both the qualitative and quantitative aspects of pain. The MPQ is highly reliable, with test–retest correlations typically exceeding 0.85, and is widely regarded as one of the most valid tools for complex pain assessment.

5. SF-36 Health Survey

The SF-36 Health Survey, developed by the RAND Corporation in the early 1990s, is a widely used tool for assessing health-related quality of life (HRQoL) across eight domains, including physical functioning, bodily pain, general health perceptions, vitality, social functioning, and mental health [24]. The tool consists of 36 items that produce scores ranging from 0 to 100 for each domain, with higher scores indicating better quality of life. The SF-36 also provides two summary scores, the Physical Component Summary (PCS) and the Mental Component Summary (MCS), offering a comprehensive overview of a patient’s physical and mental well-being. It has been extensively validated, showing strong reliability with Cronbach’s alpha values typically above 0.80, making it a robust tool for evaluating the association of chronic conditions like chronic pain on overall quality of life. The SF-36 Health Survey was employed to assess the quality of life among elderly patients with chronic pain. This survey includes multiple domains, such as physical functioning, bodily pain, and general health perceptions, which are critical in evaluating the association of pain management approaches with our study population. For the purpose of our analysis, the SF-36 scores were collected as raw scores from each respondent. To facilitate a comparative analysis across different demographic groups and ensure consistency with broader epidemiological data, these raw scores were transformed into standardized scores. The transformation process involved converting each domain score into a 0 to 100 scale, where zero represents the worst possible health state and 100 represents the best possible health state.

This method aligns with the recommendations provided in the SF-36 manual, ensuring that our results are comparable with those from other studies utilizing this scale. The standardized scores were then used in subsequent statistical analyses to assess correlations and perform multiple regression analyses, examining the influence of various predictors on quality-of-life outcomes.

### 2.9. Data Collection Procedure

The data collection process was conducted over a three-month period, from January to March 2024, across multiple secondary healthcare settings in Riyadh, Saudi Arabia. These settings included outpatient clinics, geriatric care units, and pain management clinics within several hospitals. The process was designed to ensure systematic, accurate, and ethical collection of data from elderly patients with chronic pain.

1. Participant Recruitment: Participants were identified and recruited with the assistance of nursing staff who were familiar with the patient population in each healthcare setting. The nursing staff played a crucial role in pre-screening potential participants based on the inclusion criteria. These criteria required participants to be 65 years or older, have a clinical diagnosis of chronic pain lasting three months or more, and possess the ability to communicate and provide informed consent.

Potential participants were approached by the nursing staff during their routine visits to the healthcare facility. They were provided with a brief overview of the study’s objectives and procedures, and those who expressed interest were introduced to the research team. The research team further explained the study in detail, including the purpose, the voluntary nature of participation, and the procedures involved.

2. Obtaining Informed Consent: Prior to the commencement of data collection, written informed consent was obtained from each participant. The consent process was conducted in a private setting within the healthcare facility to ensure confidentiality and provide an environment where participants could freely ask questions and express any concerns. For participants with mild cognitive impairments or those who had difficulty understanding the consent form, additional explanations were provided, and a trusted family member or caregiver was involved in the consent process if needed. The consent form included detailed information about the study’s aims, procedures, potential risks, and benefits, as well as assurances of confidentiality and the right to withdraw from the study at any time.

3. Data Collection Sessions: Data collection was conducted through face-to-face interviews, which took place in a quiet, private room within the healthcare facility to ensure a comfortable environment for the participants. The interviews were scheduled at times that were convenient for the participants, typically during their regular healthcare visits, to minimize disruption to their routine and ensure high participation rates.

Each interview session was conducted by a member of the research team, who was trained in administering the questionnaires and using the pain intensity scales. The interviews were structured but allowed for a degree of flexibility to accommodate the needs and comfort levels of the elderly participants.

4. Interview Duration and Participant Support: Each interview session lasted approximately 30 to 45 min. The researcher ensured that participants were comfortable throughout the session, offering breaks if needed and providing reassurance and support, especially when discussing sensitive topics related to pain and quality of life. Participants were encouraged to speak freely about their pain experiences and any challenges they faced in managing their condition.

### 2.10. Data Analysis

Data were analyzed using IBM SPSS Statistics (Version 28). Descriptive statistics, including means, standard deviations, frequencies, and percentages, were used to summarize the demographic data, pain intensity scores, and quality-of-life measures.

To examine the relationship between pain intensity and quality of life, Pearson correlation coefficients were calculated for the NRS, VAS, and MPQ scores against the SF-36 scores. Additionally, multiple regression analysis was performed to determine the extent to which pain intensity, as measured by the different scales, could predict quality-of-life outcomes in the elderly population.

Subgroup analyses were conducted to explore potential differences in pain intensity and quality of life based on demographic factors such as age, sex, and duration of chronic pain. The significance level for all statistical tests was set at *p* < 0.05.

Prior to conducting the multiple regression analysis, we confirmed that our data met the necessary assumptions. Linearity was assessed visually using scatterplots, independence of errors through Durbin–Watson statistic, homoscedasticity via residuals plots, and normality of errors using Q-Q plots. Variance inflation factors (VIFs) were calculated to check for multicollinearity among predictors, with all VIF values found to be below the commonly used threshold of 5, indicating no concerns of multicollinearity.

### 2.11. Ethical Considerations

This study was conducted in accordance with the ethical principles outlined in the Declaration of Helsinki. Ethical approval was obtained from the Institutional Review Board (IRB) of King Saud University (24-670). Participation in the study was entirely voluntary, and participants were assured of their right to withdraw from the study at any time without affecting their care. Confidentiality and anonymity were maintained throughout the study. All data were de-identified and stored securely in a password-protected database accessible only to the research team. The results were reported in aggregate form to prevent the identification of individual participants. Furthermore, participants were provided with information about the purpose of the study, the procedures involved, and the potential risks and benefits. Written informed consent was obtained from all participants before the commencement of data collection. Participants who required assistance with understanding the consent form were provided with appropriate support.

## 3. Results

Table 1 presents the demographic and clinical characteristics of the study population, consisting of 150 elderly patients with chronic pain. The majority of participants (49.3%) were aged between 65 and 74 years, with 34.0% in the 75–84 age group and 16.7% aged 85 and above. The sex distribution was slightly skewed, with 54.7% of the participants being female and 45.3% male. Regarding the duration of chronic pain, over half of the participants (51.3%) had been experiencing pain for more than 12 months, while 26.0% reported a duration of 3–6 months, and 22.7% reported 6–12 months. The prevalence of comorbid conditions was high, with 54.0% of participants having arthritis, 48.0% with hypertension, 42.0% with diabetes, and 29.3% with cardiovascular disease, reflecting the common health issues in this age group. In terms of pain management, the majority of participants (62.7%) were relying solely on pharmacological treatments, while 10.7% used non-pharmacological approaches, and 26.7% employed a combination of both

Table 2 presents the pain intensity scores as measured by three different scales: the Numeric Rating Scale (NRS), the Visual Analog Scale (VAS), and the McGill Pain Questionnaire (MPQ). The NRS shows a mean score of 6.7 with a standard deviation of 2.2, indicating moderate to severe pain levels among participants, with scores ranging from 2.0 to 10.0. The VAS results reflect a broader range of pain experiences, with a mean score of 62.8 and a standard deviation of 20.3, ranging from 18.5 to 97.5, which suggests significant variability in how participants perceive their pain intensity. The MPQ, which provides a more comprehensive assessment of pain, recorded a mean score of 31.7 with a standard deviation of 8.4 and a range between 14.0 and 49.0.

The correlation analysis (Table 3) revealed that all three pain intensity scales—NRS, VAS, and MPQ—were significantly negatively correlated with all quality-of-life dimensions as measured by the SF-36, with *p*-values less than 0.05. Specifically, the MPQ exhibited a strong negative correlation with physical health (r = −0.684), indicating that higher scores on the MPQ are associated with poorer physical health. Similarly, the MPQ showed moderate to strong negative correlations with mental health (r = −0.527) and overall quality of life (r = −0.614). The VAS and NRS demonstrated moderate negative correlations with these dimensions, highlighting that while the scales are effective in capturing the association of pain with quality of life, the extent of correlation varies, likely due to the different aspects of pain each scale measures.

Table 4 offers a robust multiple regression analysis illustrating the significant factors that predict the quality of life in elderly patients dealing with chronic pain. The model demonstrates strong predictive validity with an R^2^ of 0.71 and an adjusted R^2^ of 0.68, indicating that approximately 68% of the variance in quality of life can be explained by the predictors in the model. Key predictors include pain intensity scales such as the Numeric Rating Scale (NRS, β = −0.52), Visual Analog Scale (VAS, β = −0.55), and McGill Pain Questionnaire (MPQ, β = −0.61), all showing significant negative associations with quality of life (*p* < 0.001). Demographic factors like age (β = −0.18, *p* = 0.028) and sex (β = −0.22, *p* = 0.023) also notably contribute, indicating that older age and being male are associated with poorer quality of life. Comorbid conditions—particularly cardiovascular disease (β = −0.17, *p* = 0.002) and arthritis (β = −0.14, *p* = 0.005)—further associate with quality of life adversely. The significant F-statistic (36.2) with a *p*−value < 0.001 confirms the overall model’s statistical significance, reinforcing the critical need for a holistic approach to managing chronic pain in the elderly that addresses these diverse factors.

Table 5 provides a comprehensive breakdown of quality-of-life scores among elderly patients with chronic pain, delineating the association of different pain management strategies across varied demographic factors. The analysis reveals a consistent pattern: non-pharmacological approaches consistently yield the highest quality-of-life scores across all demographic groups, with mean scores notably higher in physical and mental health domains compared to pharmacological and combined approaches. For instance, in the overall category, non-pharmacological methods resulted in an overall quality-of-life score of 53.5, compared to 46.5 for pharmacological only and 51.3 for combined approaches, with statistically significant differences (*p*-values < 0.024).

The association of these treatment strategies also varies with age, sex, and duration of chronic pain. Older age groups (85+) show a pronounced decrease in quality-of-life scores across all treatment modalities, highlighting the challenge of managing chronic pain effectively in this demographic. Similarly, males generally report lower quality-of-life scores than females, suggesting potential differences in pain perception or treatment efficacy between genders. Notably, longer durations of chronic pain (>12 months) correlate with lower quality-of-life scores, emphasizing the cumulative detrimental effect of prolonged pain.

## 4. Discussion

This study aimed to assess the association of pain intensity scale administrations on quality of life in elderly patients with chronic pain, providing insights into the complex relationship between pain assessment methodologies and patient-reported outcomes. The findings reveal significant correlations between pain intensity and quality of life, with variations observed across different assessment tools and demographic factors.

### 4.1. Interpretation of Pain Scale Comparisons

The results demonstrate that all three pain intensity scales—NRS, VAS, and MPQ—showed significant negative correlations with quality-of-life dimensions as measured by the SF-36. However, it is crucial to interpret these comparisons cautiously, as each scale captures different aspects of the pain experience. The MPQ exhibited a significant correlation with physical health (r = −0.684), mental health (r = −0.527), and overall quality of life (r = −0.614). This finding aligns with the comprehensive nature of the MPQ, which assesses not only pain intensity but also its qualitative aspects and association with various domains of life [25,26].

The significant correlation of the MPQ with quality-of-life measures compared to the NRS and VAS can be attributed to its multidimensional approach. While the NRS and VAS primarily focus on pain intensity, the MPQ incorporates sensory, affective, and evaluative dimensions of pain [27]. This comprehensive assessment may better reflect the complex pain experience of elderly patients, capturing nuances that simpler scales might miss. However, it is important to note that the strength of correlation does not necessarily indicate the superiority of one scale over others, as each has its own merits and practical applications in clinical settings [28,29].

The differences in correlation strengths between scales highlight the importance of choosing appropriate assessment tools based on the specific needs of the patient population and the clinical context. For instance, while the MPQ provides a more detailed pain assessment, its length and complexity may make it less practical for routine use in some clinical settings, particularly with elderly patients who may have cognitive limitations or fatigue easily [30].

### 4.2. Factors Influencing Quality of Life in Elderly Patients with Chronic Pain

The multiple regression analysis revealed several significant predictors of quality of life, including pain intensity, age, sex, chronic pain duration, and comorbid conditions. These findings underscore the multifaceted nature of the relationship between chronic pain and quality of life in the elderly population.

The negative association between age and quality of life (β = −0.18, *p* = 0.028) aligns with previous research indicating that older individuals often experience a decline in quality of life due to various age-related factors, including increased prevalence of chronic conditions and functional limitations [31]. The sex difference observed (β = −0.22, *p* = 0.023), with males reporting lower quality-of-life scores, warrants further investigation. This finding contrasts with some previous studies that have reported lower quality-of-life scores in females with chronic pain [32,33]. The discrepancy could be due to cultural factors, differences in pain coping strategies, or variations in healthcare-seeking behaviors between genders in the study population [34,35].

The significant association of chronic pain duration on quality of life (β = −0.27, *p* = 0.011) highlights the cumulative effect of prolonged pain experiences on overall well-being. This finding emphasizes the importance of early intervention and effective long-term management strategies to mitigate the negative association of chronic pain with quality of life [36].

The influence of comorbid conditions, particularly cardiovascular disease (β = −0.17, *p* = 0.002) and arthritis (β = −0.14, *p* = 0.005), on quality of life underscores the complex interplay between chronic pain and other health conditions in the elderly. These results align with previous research demonstrating the compounding effect of multiple chronic conditions on health-related quality of life [37].

### 4.3. Comparative Effectiveness of Pain Management Approaches

The analysis of quality-of-life scores based on pain management approaches revealed that non-pharmacological methods consistently yielded higher scores across all demographic groups. This finding is particularly noteworthy, as it challenges the traditional reliance on pharmacological interventions as the primary approach to pain management in elderly patients.

The superior outcomes associated with non-pharmacological approaches could be attributed to several factors. Firstly, these methods often address the multidimensional nature of pain, incorporating physical, psychological, and social aspects of pain management [38]. Non-pharmacological approaches may have fewer side effects compared to medications, which is particularly relevant for elderly patients who are more susceptible to adverse drug reactions [39]. These approaches often involve active patient participation, which may enhance feelings of self-efficacy and control over pain management, positively associating quality of life [40].

However, it is important to note that the effectiveness of pain management approaches may vary based on individual patient characteristics, pain etiology, and severity. The finding that combined approaches (pharmacological and non-pharmacological) also showed improved quality-of-life scores compared to pharmacological methods alone suggests that an integrated, multimodal approach to pain management may be beneficial for many elderly patients [41].

### 4.4. Relationship Between Pain Assessment and Quality of Life

The study results raise important questions about the relationship between pain assessment methodologies and quality-of-life outcomes. While the findings demonstrate that quality-of-life scores vary depending on the pain scale used, it would be premature to conclude that quality of life is directly dependent on the type of scale used to assess pain [42]. Instead, these variations likely reflect the different aspects of the pain experience captured by each scale and how these aspects relate to overall quality of life [43].

The MPQ’s significant correlation with quality-of-life measures may indicate that a more comprehensive pain assessment provides a better reflection of how pain associations various life domains. This suggests that quality of life in elderly patients with chronic pain is influenced not just by pain intensity but by the broader pain experience, including its sensory and affective components [44,45,46].

### 4.5. Limitations of the Study

Limitations of this study include its cross-sectional design, which precludes the establishment of causal relationships between pain intensity and quality of life. The ability to draw definitive conclusions about how changes in pain over time affect quality of life is restricted. Additionally, the sample was drawn from secondary healthcare settings in Riyadh, Saudi Arabia, which may limit the generalizability of findings to other cultural contexts or healthcare systems. The reliance on self-reported measures for both pain intensity and quality of life introduces the potential for recall bias, particularly in an elderly population where cognitive impairments may be present. Furthermore, the study did not account for all possible confounding factors that could influence the relationship between pain and quality of life, such as specific pain medications, detailed treatment histories, or comprehensive psychosocial factors.

### 4.6. The Implications of This Study for Nursing Practice

The implications of this study for nursing practice are substantial and multifaceted. Firstly, nurses should prioritize the use of comprehensive pain assessment tools, such as the McGill Pain Questionnaire, alongside simpler scales like the NRS and VAS, to capture the full complexity of the pain experience in elderly patients. The strong correlation between pain intensity and quality of life underscores the need for regular, thorough pain assessments as a routine part of elderly patient care. Secondly, the findings support the integration of non-pharmacological approaches into pain management strategies, as these were associated with higher quality-of-life scores. Nurses can play a crucial role in educating patients about these approaches and facilitating their implementation. Thirdly, the study highlights the importance of individualized, patient-centered care that takes into account factors such as age, sex, comorbidities, and duration of chronic pain. Nurses should consider these factors when developing and implementing pain management plans. Lastly, the relationship between pain duration and quality of life emphasizes the need for early intervention and ongoing management of chronic pain to prevent long-term decline in quality of life.

## 5. Conclusions

This study provides compelling evidence for the significant association of pain intensity on quality of life in elderly patients with chronic pain. It underscores the complex, multidimensional nature of chronic pain and its effects on various aspects of well-being in older adults. The findings highlight the need for a holistic approach to pain assessment and management that goes beyond pharmacological interventions. By adopting comprehensive assessment strategies, integrating non-pharmacological approaches, and providing individualized care, nurses can play a pivotal role in improving the quality of life for elderly patients with chronic pain. This study contributes to the growing body of evidence supporting patient-centered, multimodal approaches to pain management in geriatric care and emphasizes the critical role of nursing in addressing the challenges of chronic pain in an aging population.

## Figures and Tables

**Table 1:** Demographic and clinical characteristics of the study population.

Characteristic	Frequency (n = 150)	Percentage (%)
Age Group		
65–74 years	74	49.3
75–84 years	51	34.0
85+ years	25	16.7
Sex		
Male	68	45.3
Female	82	54.7
Duration of Chronic Pain		
3–6 months	39	26.0
6–12 months	34	22.7
>12 months	77	51.3
Comorbid Conditions		
Diabetes	63	42.0
Hypertension	72	48.0
Cardiovascular Disease	44	29.3
Arthritis	81	54.0
Current Pain Management		
Pharmacological only	94	62.7
Non-pharmacological only	16	10.7
Combined approaches	40	26.7

n: number of participants; %: percentage of the total sample.

**Table 2:** Pain intensity scores across different scales.

Pain Intensity Scale	Mean Score	Median Score	Standard Deviation	Range (Min–Max)
Numeric Rating Scale (NRS)	6.7	7	2.2	2.0–10.0
Visual Analog Scale (VAS)	62.8	60	20.3	18.5–97.5
McGill Pain Questionnaire (MPQ)	31.7	32	8.4	14.0–49.0

NRS: Numeric Rating Scale; VAS: Visual Analog Scale; MPQ: McGill Pain Questionnaire.

**Table 3:** Correlation between pain intensity and quality-of-life scores.

Pain Intensity Scale	Physical Health (SF-36)	Mental Health (SF-36)	Overall Quality of Life (SF-36)
Numeric Rating Scale (NRS)	−0.576 *	−0.437 *	−0.525 *
Visual Analog Scale (VAS)	−0.617 *	−0.459 *	−0.553 *
McGill Pain Questionnaire (MPQ)	−0.684 *	−0.527 *	−0.614 *

* *p* < 0.05; SF-36: a health survey used to measure quality of life.

**Table 4:** Multiple regression analysis of factors predicting quality of life.

Predictor Variables	B	Standard Error	Beta	*p*-Value
Numeric Rating Scale (NRS)	−0.85	0.14	−0.52	<0.001
Visual Analog Scale (VAS)	−0.039	0.012	−0.55	<0.001
McGill Pain Questionnaire (MPQ)	−0.243	0.065	−0.61	<0.001
Age	−0.115	0.048	−0.18	0.028
Sex	−1.37	0.59	−0.22	0.023
Chronic Pain Duration	−0.091	0.033	−0.27	0.011
Diabetes	−0.058	0.021	−0.15	0.010
Hypertension	−0.030	0.019	−0.09	0.084
Cardiovascular Disease	−0.101	0.032	−0.17	0.002
Arthritis	−0.083	0.028	−0.14	0.005

Model Fit: R^2^ = 0.65, adjusted R^2^ = 0.62, F-statistic = 27.3, *p*-value = <0.001; B: unstandardized regression coefficient; Beta: standardized regression coefficient; *p*-value: values less than 0.05 are typically considered statistically significant.

**Table 5:** Comparison of quality-of-life scores based on pain management approaches.

Demographic Factor	Pain Management Approach	Physical Health (SF-36)	Mental Health (SF-36)	Overall Quality of Life (SF-36)	*p*-Value
Overall	Pharmacological only	44.8	48.3	46.5	0.019
	Non-pharmacological only	52.6	54.9	53.5	0.024
	Combined approaches	49.7	53.1	51.3	0.021
Age Group					
65–74	Pharmacological only	46.2	50.0	48.1	0.030
	Non-pharmacological only	54.0	56.3	55.1	0.022
	Combined approaches	51.5	55.0	53.3	0.026
75–84	Pharmacological only	43.5	47.0	45.2	0.035
	Non-pharmacological only	53.2	55.6	54.4	0.028
	Combined approaches	50.1	52.8	51.4	0.032
85+	Pharmacological only	41.8	45.5	43.6	0.040
	Non-pharmacological only	51.9	54.2	53.0	0.034
	Combined approaches	48.5	50.9	49.7	0.038
Sex					
Male	Pharmacological only	43.9	47.1	45.5	0.033
	Non-pharmacological only	51.7	53.8	52.8	0.027
	Combined approaches	49.0	51.5	50.3	0.029
Female	Pharmacological only	45.7	49.5	47.6	0.020
	Non-pharmacological only	53.4	56.1	54.8	0.018
	Combined approaches	50.4	54.3	52.4	0.023
Chronic Pain Duration					
<6 months	Pharmacological only	46.0	49.8	47.9	0.025
	Non-pharmacological only	54.8	57.0	55.9	0.015
	Combined approaches	52.0	55.5	53.8	0.020
>12 months	Pharmacological only	42.4	46.7	44.6	0.038
	Non-pharmacological only	50.5	52.9	51.7	0.028
	Combined approaches	47.9	51.1	49.5	0.030

SF-36: a health survey used to measure quality of life.

## Data Availability

All data are available within the manuscript.
